# Bridging Viral Glycobiology and Lectin Biotechnology for Antiviral and Diagnostic Strategies

**DOI:** 10.1021/acsbiomedchemau.5c00115

**Published:** 2025-07-25

**Authors:** Benildo Sousa Cavada, Vinicius Jose Silva Osterne, Messias Vital Oliveira, Wandemberg Paiva Ferreira, Cornevile Correia Neto, Kyria Santiago Nascimento, Vanir Reis Pinto-Junior

**Affiliations:** † Department of Biochemistry and Molecular Biology, BioMol-Lab, Federal University of Ceara, Fortaleza 60020-181, CE, Brazil; ‡ Department of Physics, Federal University of Ceara, Fortaleza 60020-181, CE, Brazil

**Keywords:** lectins, viruses, viral glycoproteins, antiviral agents, glycan shield, diagnostic tools, biosensors, bioengineering

## Abstract

Lectins, proteins that reversibly bind specific glycan motifs, offer dual utility as molecular probes or inhibitors of virus–host interactions. This review explores the molecular interactions between lectins and viral envelope glycoproteins, emphasizing their applications as antiviral agents and diagnostic tools. Enveloped viruses, such as HIV, Influenza, Herpesviruses, and Coronaviruses, exhibit dense glycosylation on their surface proteins, forming a glycan shield rich in high-mannose and complex glycans crucial for viral processes and immune evasion. Lectins exploit these glycan shields by selectively targeting conserved glycosylation sites on key viral proteins like gp120 (HIV), hemagglutinin (Influenza), spike (SARS-CoV-2), and glycoprotein D (HSV), thereby interfering with viral entry. Potent inhibitory activity across diverse virus families has been demonstrated for natural lectins such as griffithsin (GRFT), cyanovirin (CV–N), and banana lectin (BanLec), with novel fungal and algal lectins continually expanding the list. Concurrently, lectin-based biosensors utilizing electrochemical, plasmonic, and microfluidic platforms, often enhanced by nanomaterials or aptamers, enable sensitive and specific detection of glycosylated viral targets. Despite challenges including potential immunogenicity and production scalability, ongoing bioengineering efforts aim to refine lectin specificity, reduce toxicity, and enhance overall functionality. These collective advances showcase the role of lectins as versatile molecular tools for the detection, inhibition, and mechanistic study of viral pathogens.

## Graphical abstract

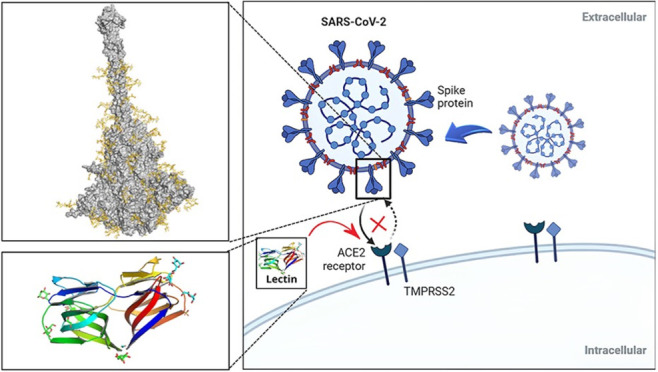

## 1 Introduction

Viruses remain among the most impactful infectious agents worldwide, causing high morbidity and mortality across the world. It is estimated that influenza viruses infect over one billion people annually with up to half a million deaths attributed to seasonal forms of the disease. Since its emergence in 2019, the devastating impact of COVID-19, marked by over 700 million cases and nearly 7 million deaths, has sharply accelerated virology research.^1,2^ In addition to these agents, other viruses, such as HIV, herpesviruses, and various emerging pathogens, continue to pose persistent challenges to public health, especially in light of the rise of antiviral-resistant variants.^3,4^

A key element in understanding enveloped viruses lies in their surface glycoproteins, membrane-anchored structures that play essential roles in host cell attachment, membrane fusion, and immune evasion. Glycoproteins are generated through glycosylation, a post-translational modification that attaches carbohydrate chains to specific amino acid residues. In viruses, glycosylation mainly occurs through N-linked and O-linked pathways: N-glycans attach to asparagine residues within the Asn-X-Ser/Thr motif, while O-glycans bind directly to serine or threonine.^5^ Many viral surface glycoproteins are extensively N-glycosylated, often presenting high-mannose structures, though hybrid and complex glycans are also found, depending on the virus.^6^ These carbohydrate motifs often represent conserved viral features, providing potential targets for therapeutic intervention.^7^ Viruses, including HIV-1, Influenza, SARS-CoV-2, and herpesviruses, rely heavily on these glycan shields to evade host immune responses. The dense glycosylation patterns mimic host glycoconjugates, making viral surfaces less recognizable to neutralizing antibodies. Recent studies have shown that certain glycan-binding proteins, such as *Streptococcal* Siglec-like lectin (SLBR-N) and the plant-derived jacalin, can modulate infectivity, and antibody susceptibility in HIV-1 by binding to O-glycans on the viral envelope glycoproteins.^8^

Among such glycan-binding proteins are the lectins, naturally occurring proteins found across a wide range of organisms, including plants, algae, bacteria, and animals. They are characterized by the presence of one or more carbohydrate-recognition domain (CRD) in their structures. CRDs are defined as noncatalytic domains that specifically and reversibly bind to carbohydrates without inducing covalent modifications.^9,10^ Unlike antibodies, which typically recognize protein epitopes, lectins interact with glycan structures on glycoproteins and glycolipids, making them promising candidates for therapeutic and diagnostic applications, particularly in the field of virology.^7,11^ Indeed, numerous studies demonstrate that various natural and engineered lectins can inhibit viral infection. By binding to viral surface glycoproteins, they interfere with essential steps of the viral life cycle, such as host cell attachment, membrane fusion, and subsequent spread. This mechanism holds potential against emerging or resistant viral strains due to the relatively conserved nature of key glycosylation sites across different variants.

Furthermore, the utility of lectins extends beyond direct viral inhibition, driven by ongoing biotechnological innovation. Strategies such as protein engineering and the creation of novel fusion constructs (e.g., lectibodies) seek to optimize lectins for therapeutic use. Their specific glycan-binding properties are also being exploited for the development of molecular diagnostic assays and biosensors. Additionally, investigations into their interactions with the immune system explore their potential as vaccine adjuvants.^12^

Despite this potential and the noted advances, several limitations still hinder widespread clinical translation, including persistent challenges with immunogenicity, dose-dependent cytotoxicity, and scalability of production. Thus, a deeper understanding of their structure–function relationships and mechanisms of action is essential to fully harness their therapeutic potential. In this context, we understand that the convergence of viral glycobiology and lectin applications marks an important shift from lectins being confined to academic, wet-lab studies toward their development as practical antiviral biotechnological tools. A comprehensive understanding of the glycosylation profiles of different virus families reveals not just defensive structures but also conserved vulnerabilities that can be systematically exploited. This review will therefore critically examine the structure–function relationships of viral glycoproteins and their interaction with exogenous lectins. We will synthesize the current state of knowledge to build a case for moving beyond the discovery of individual antiviral lectins toward the rational design of next-generation glycan-targeting biologics for broad-spectrum therapeutic and diagnostic applications.

## 2 Virus Structure and Glycobiology

### 2.1 Influenza Virus (*Orthomyxoviridae*)

The *Orthomyxoviridae* family comprises seven genera of RNA viruses, notably Influenzavirus A (*Alphainfluenzavirus*), B (*Betainfluenzavirus*), and C (*Gammainfluenzavirus*).^13^ Influenza A and B viruses cause influenza (or flu), an infectious respiratory disease in humans while influenza C can cause flu-like symptoms, but with less intensity and a higher infection rate in children.^14,15^ Influenza A and B result in seasonal epidemics, with approximately 1 billion infections and 300–500 thousand deaths per year, despite vaccination campaigns.^13^ Symptoms of influenza include high fever, headache, malaise, weakness, dry cough, sore throat, and runny nose. Older adults are more susceptible to the disease and may develop more severe respiratory symptoms, such as pneumonia, requiring hospitalization.^16^ History records four influenza A-related pandemics: the Spanish Flu (H1N1) in 1918, the Asian Flu (H2N2) in 1957, the Hong Kong Flu (H3N2) in 1968, and the Swine Flu A­(H1N1)­pdm09 in 2009.^17^

Influenza viruses are enveloped and possess an RNA genome consisting of eight segments. The genome contains information for various proteins, such as RNA polymerase, viral nucleoprotein (NP), matrix protein (M1), membrane protein (M2), nonstructural protein (NS1), nuclear export protein (NEP), and viral glycoproteins like hemagglutinin (HA), all responsible for virus entry into the host cell, as well as neuraminidase (NA), which plays a role in the viral release and cellular specificity.^13,18^ The surface glycoproteins of influenza viruses, hemagglutinin (HA) and neuraminidase (NA), are primary targets of protective antibodies, vaccines, and antiviral therapies (Figure 1a). These glycoproteins also exhibit antigenic variations that can be used to classify viruses into subtypes that can be identified by the acronym HxNy, where x and y indicate specific variants of these proteins.^19^ Membrane type I glycoprotein HA is crucial for viral infection as it recognizes specific glycosylated receptors on host cells.^20^

**Figure 1:**
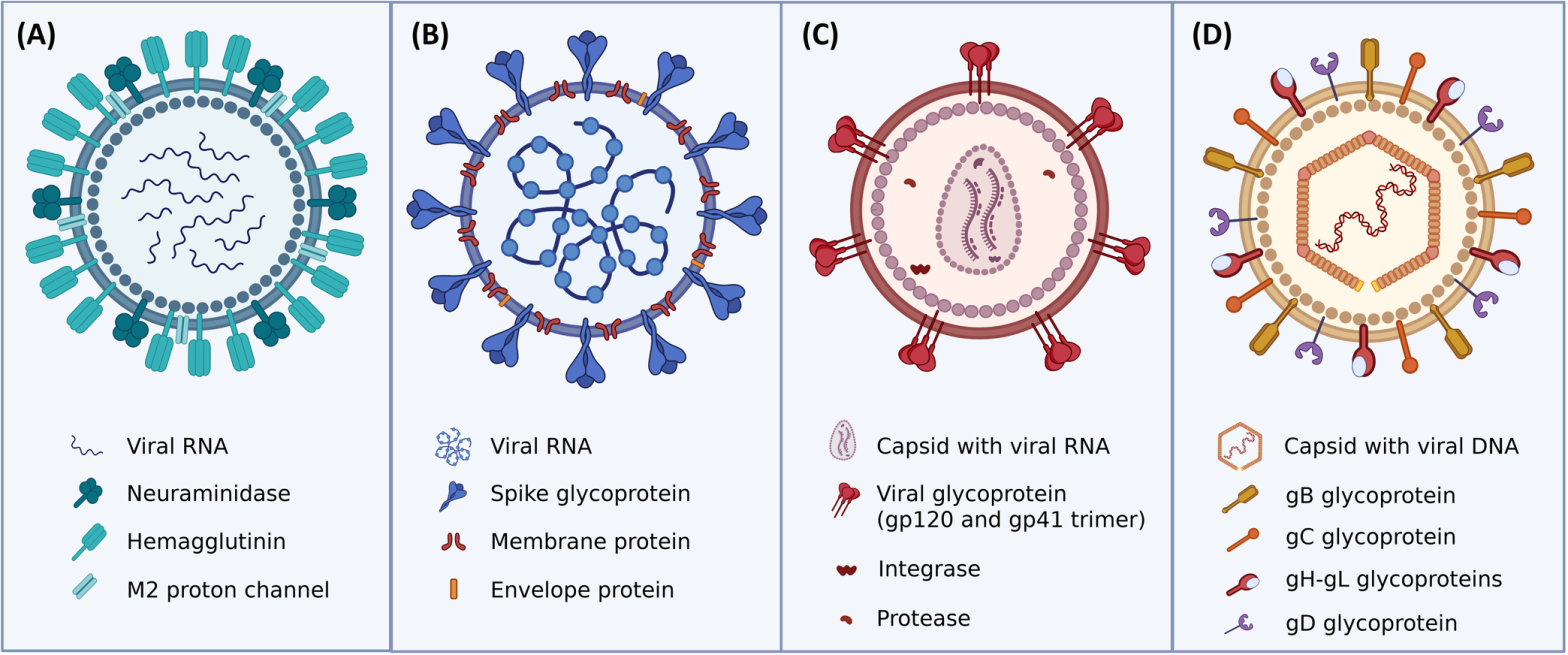
General structure of virions, highlighting the compartmentalization of the genome and surface proteins. (A) Influenza A virus (IAV); (B) Coronavirus (CoV); (C) Human Immunodeficiency Virus (HIV); (D) *Herpes Simplex* Virus (HSV).

HA is extensively glycosylated, with glycan sites and patterns differing between strains due to mutations. In the stem region, glycosylation mainly influences protein folding, structural stability, and membrane fusion. In contrast, glycosylation in the globular head region plays a more direct role in receptor binding and evasion of the host immune response.^21–24^ N-glycans in the stem of HA tend to be more conserved among virus strains, while those in the head are more variable in terms of position, quantity, and composition.^25,26^ Evolutionary analyses indicate that glycosylation in the head has increased in human-adapted strains, often featuring a higher proportion of high-mannose N-glycans compared to avian strains.^26–28^

Influenza virus glycoproteins have two main types of N-glycans, high-mannose and complex; the proportion of which directly affects virus–host interaction.^29–31^ High-mannose N-glycans have multiple mannose residues (2 to 6) linked to the common trimannoside core (Man_3_GlcNAc_2_) and are poorly processed in the Golgi apparatus.^11,32^ These glycans are best recognized by cellular receptors that can facilitate the infection process, but are also best recognized by innate immunity receptors, such as MBL (mannose-binding lectin) and DC-SIGN, facilitating complement activation and viral elimination.^33^ On the other hand, complex glycans have branches with residues of N-acetylglucosamine, galactose, sialic acid and fucose.^11^ These glycans confer protection against the immune response by masking epitopes on surface proteins (hemagglutinin and neuraminidase) and making recognition by neutralizing antibodies difficult.^31^ The proportion of these glycans influences the balance between immunogenicity and immune evasion. Viruses with more high-mannose N-glycans are more easily recognized by innate immunity, while those with a predominance of complex glycans evade the adaptive immune response more efficiently.^30,32^

Detailed glycomic analyses exemplify this variability. The pH1N1 (A/California/07/2009) and H3N2 (A/Victoria/361/2011) viruses of human origin exhibit a predominance of high-mannose and complex N-glycans, with significant site-specific occupancy variation. H1 typically has ∼ 6 N-glycosylation sites with a higher proportion of high-mannose N-glycans at sites N94, N278, and N289 and a higher proportion of complex glycans at N21, N33, and N483. H3 is highly glycosylated with 13 glycosylation sites. Of these sites, N22, N38, N63, N126, N246, and N483 exhibit a predominance of complex glycans, while N133, N165, and N285 have a predominance of high-mannose glycans. At sites N45 and N144, glycosylation may exhibit equal abundance for both glycan classes, or the site may be unoccupied. No glycans were detected at the N122 site. Strains originating from birds, such as A/Vietnam/1203/2004 (H5N1), A/Taiwan/2/2013 (H6N1), A/Shanghai/2/2013 (H7N9), and A/Jiangxi-Donghu/346/2013 (H10N8), exhibited a profile with a lower number of glycosylations and a stronger predominance of complex N-glycans.^28^

Further examples include the A/NewCaledonia/20/1999 (H1N1) virus, possessing eight occupied N-glycosylation sites, four in the stem (N28, N40, N304, and N498) and four in the head (N71, N104, N142, and N177). Analyses have revealed the presence of high-abundance complex and hybrid glycans with high-mannose N-glycans at N142 and N177. Fucosylation was observed in most detected glycans, particularly core fucosylation. N498 displayed bulky complex glycans. Glycans at N142 and N498 frequently exhibited fucosylation and sialylation, resulting in a wide diversity of glycans at these two sites.^34^ Analysis of HA glycosylation from candidate recombinant vaccines, such as NIBRG-121xp and NYMC-X181A derived from A/California/07/2009, revealed distinct profiles with nine occupied N-glycosylation sites identified. The N27/N28 site presented the simplest glycan composition with the complex biantennary glycan GalGlcNAc2Man3GlcNAc2. The N40, N136, N293, and N498 sites showed complex bi- and triantennary N-glycans with predominant core fucosylation and tetra-antennary N40. The N104 and N304 sites exhibited high-mannose N-glycans, with N304 chains varying between 4 and 8 mannosyl residues. The N40, N104, and N490 sites presented hybrid triantennary N-glycans. The N136 site, added by mutation, showed the highest heterogeneity, presenting complex, hybrid, and high-mannose N-glycans, which could be bi-, tri-, or tetra-antennary, and were sulfated and fucosylated.^35^

The second one is neuraminidase (NA), which is a type II membrane glycoprotein that is dependent on Ca2+. The activity of this enzyme allows the movement of the virion toward susceptible cells.^36^ NA assembles into tetramers, with each polypeptide comprising four distinct structural domains: the cytoplasmic tail, the transmembrane region, a stem, and the catalytic head.^37–39^ Alterations in NA glycosylation generally have a less significant impact on viral fitness than those in HA. NA of the 1918 pandemic H1N1 influenza virus had four glycosylation sites on the stem (N50, N58, N63, and N68) and three on the globular head (N88, N146, and N235). Over the years, only the position of glycosylation sites has changed, not the quantity of glycosylation. These studies have also shown that the NA of other influenza virus subtypes, such as H3N2 and Influenza B, do not exhibit clear trends of increased glycan counts.^33^ Analysis of NA from the aforementioned NIBRG-121xp and NYMC-X181A vaccines revealed the presence of six occupied consensus N-glycosylation sites. Bi- and triantennary N-glycans representing high-mannose and complex glycans types were observed at sites N50, N68, and N235. Site N58 contained high-mannose N-glycans of the Man5–9GlcNAc2 type. A single complex glycan of the Gal2GlcNAc2Man3GlcNAc2 type was found at site N386. Site N146 exhibited high heterogeneity, including complexes, hybrids, and high-mannose structures with different degrees of branching and modifications, such as sulfation and fucosylation. In smaller proportions, tetra-antennary structures containing N-acetylglucosamine linked to the core mannose structure were found.^35^ NA glycosylation can modulate its enzymatic activity and thereby influence viral replication efficiency, with specific changes implicated in recent zoonotic transmissions from pigs.^31,40,41^

These studies demonstrate the significance of analyzing the glycosylation of HA and NA in understanding the biology of the influenza virus and in the development of more effective therapeutic strategies and vaccines.

### 2.2 SARS-CoV-1, SARS-CoV-2 e MERS-CoV (*Coronaviridae*)

The *Coronaviridae* family comprises a diverse group of enveloped, positive-sense single-stranded RNA viruses.^42^ While many coronaviruses (CoVs) cause mild respiratory or digestive illness, the emergence of highly pathogenic *betacoronaviruses* in the 21st century, namely Severe Acute Respiratory Syndrome Coronavirus (SARS-CoV-1), Middle East Respiratory Syndrome Coronavirus (MERS-CoV), and SARS-CoV-2, has resulted in significant human morbidity and mortality, primarily through severe respiratory disease.^43,44^ The rapid evolution of SARS-CoV-2, leading to variants of concern, continues to pose challenges for therapeutic and vaccination strategies.

Coronavirus genomes are notably large for RNA viruses (∼30 kb) and typically encode nonstructural replicase proteins (from ORF1a/1b) and the main structural proteins: spike (S), envelope (E), membrane (M), and nucleocapsid (N). Structurally, coronaviruses are roughly spherical virions (∼100–150 nm diameter) with a host-derived lipid envelope embedding the S, M, and E proteins. The characteristic surface spikes are formed by the S protein, while a helical nucleocapsid (N protein complexed with RNA) resides within (Figure 1b).^45,46^

The spike protein (S) is a class I fusion glycoprotein and the most prominent structure on the virion’s surface. It assembles into trimers, forming the spikes responsible for host cell entry.^47^ Each S monomer (∼1200–1300 amino acids) has two functional subunits: S1, which contains the receptor-binding domain (RBD), and S2, which mediates membrane fusion. The S protein from CoV binds to the host cell’s receptor to initiate entry. Structurally, the trimeric S protein extends outward from the virion, with the S1 subunit forming the globular head that contains the RBD, and in coronaviruses, a receptor-binding motif (RBM) within it, while the S2 subunit forms the stalk that anchors the complex in the viral envelope. The S protein is the primary determinant of host range and tissue tropism, and it is also the principal target of neutralizing antibodies and vaccines.

Other proteins include the membrane protein (M) which is the most abundant structural protein shaping the virion and allowing assembly by organizing the ER-Golgi membranes and interacting with other structural proteins. The M protein features a glycosylated N-terminal ectodomain.^48^ The envelope protein (E) is crucial for virus production and pathogenesis^49^ and the nucleocapsid protein (N) is essential for genome packaging and virion assembly through interactions with the M protein. N protein also modulates host immune responses.^50,51^

The spike glycoproteins of these three pathogenic *betacoronaviruses* are heavily glycosylated, a feature that profoundly influences their biology. The S proteins fold and trimerize in the endoplasmic reticulum and traverse the secretory pathway through the ER-Golgi, acquiring N-linked glycans at numerous sequons (Asn-X-Ser/Thr sequons) along the polypeptide. N-linked glycosylation of coronavirus spikes is extensive. Specifically, SARS-CoV-1 S carries ∼23 N-glycosylation sites, MERS-CoV S ∼23 sites, and SARS-CoV-2 S ∼22 sites per monomer.^52^ Thus, a substantial portion of the spike’s mass consists of sugars. The attached glycans are predominantly complex-type and high-mannose-type oligosaccharides, added by host cell enzymes. Glycosylation is generally similar in overall extent for these viruses. However, SARS-CoV-2 has one fewer N-glycan site than its predecessor and, hence, a slightly sparser glycan coat.^52–54^ Despite this small reduction in glycan count, SARS-CoV-2 turned out to be more infectious and transmissible than SARS-CoV-1, indicating that factors beyond sheer glycan number, e.g., receptor affinity or cleavage activation, play major roles in pathogenicity.^55^

These extensive spike glycans serve multiple functions. (1) Immune evasion – the dense coat of host-derived sugars on the spike protein can mask underlying viral peptide epitopes from recognition by the host immune system.^56^ Because these glycans are added by host enzymes, they are chemically identical to “self” glycans found on human glycoproteins and thus poor targets for the immune system. By sterically covering large portions of the protein surface, glycans hide conserved protein regions that might otherwise be targeted by neutralizing antibodies.^57^ For example, glycans surrounding the receptor-binding site and fusion peptide help cloak those vulnerable regions. The flip side is that not all epitopes are glycosylated. However, this shield is incomplete; neutralizing antibodies can find unglycosylated holes, such as the RBM on SARS-CoV-2 RBD, which is largely unglycosylated, allowing receptor engagement. Additionally, some potent antibodies (e.g., S309) recognize conserved epitopes comprising both glycan (e.g., at N343) and peptide components, and can even neutralize related viruses like SARS-CoV-1.^47^ (2) Assisting viral entry - Glycans on the spike protein facilitate entry by (i) mediating initial attachment to host cell lectins or glycan-binding receptors like DC-SIGN and heparan sulfate, concentrating virus at the cell surface;^47,58,59^ (ii) enhancing infection;^60,61^ and (iii) modulating spike-receptor interaction.^62,63^

Detailed glycomic analyses reveal significant differences in the types of *N*-glycans decorating each spike. For comparison, when the spikes are produced in the same cell type, MERS-CoV S tends to retain a larger fraction of high-mannose glycans, whereas SARS-CoV-1 and SARS-CoV-2 S proteins are more extensively processed to complex-type glycans.^64^ In one analysis of the S1 subunit glycome, MERS-CoV S1 had a significantly higher proportion of Man5–9 glycans, indicating less trimming in the Golgi, while the S1 of both SARS-CoV-1 and SARS-CoV-2 had most glycans processed to complex forms with sialic acid and fucose. SARS-CoV-2 S showed the highest level of complex glycans and very low levels of hybrid-type glycans. These differences could result from intrinsic sequence differences (e.g., accessibility of N-sites due to local protein folding affecting enzyme access) or potentially from variations in protein processing kinetics.^64,65^ Biologically, a higher high-mannose content on MERS-CoV S might influence epitope presentation or interactions with sialic acid receptors (which MERS-CoV S is known to bind).^66,67^ By contrast, SARS-CoV-2 S, having predominantly complex glycans, more closely mimics host cell-surface glycoproteins, possibly suggesting another immune evasion strategy. It is worth noting that all three spikes still have a mixture of glycan types and that the overall glycan shield coverage is broadly similar. Nevertheless, the noted fine distinctions likely influence how each virus engages lectins or is sensed by host lectin receptors.^68^

In addition to N-glycans, O-glycosylation on coronavirus spike proteins has attracted attention. O-glycans are generally less prevalent on coronavirus S than N-glycans, but they do occur. A comparative study of recombinant S1 subunits found that SARS-CoV-2 S1 had several O-glycosylation sites, particularly clustered near the unique polybasic (PRRA) furin cleavage site in the S1/S2 junction region, more so than observed for SARS-CoV-1 and MERS-CoV S1 in that region. Evidence suggests SARS-CoV-2 has more O-glycans proximal to the cleavage site than the other β-coronaviruses.^69^ These O-glycans, despite potentially low occupancy per site, might modulate the accessibility or processing of the cleavage site by host proteases. Overall, O-glycosylation levels on coronavirus spikes are much lower than those of N-glycosylation and often heterogeneous, but they add another layer of glycan decoration. MERS-CoV, SARS-CoV-1, and SARS-CoV-2 all have some O-glycosylation on their spikes, but current data indicate that SARS-CoV-2′s spike might carry O-glycans in functionally relevant positions, e.g., around the polybasic cleavage site, more so than SARS-CoV-1.^52^ The functional impact of these O-linked sugars is still being investigated, but one hypothesis is that they could influence cleavage efficiency or local charge, or they could provide additional immune-evasive shielding in a crucial region of the spike.

Aside from spike, the other structural proteins show modest glycosylation. As stated before, the M protein has a single N-glycan, and the E protein is essentially unglycosylated. These differences in glycosylation patterns across SARS-CoV-1, SARS-CoV-2, and MERS-CoV spikes may subtly affect how these viruses interact with the host.^53^

### 2.3 HIV (Retroviridae)

The *Retroviridae* family consists of enveloped viruses with RNA genomes that primarily infect humans and vertebrate animals. During their replication, they transcribe their RNA into an intermediate form of DNA, which explains why they are called retroviruses. The human immunodeficiency virus (HIV) is an example of a retrovirus that has received significant public health attention.^70^ HIV is a lentivirus with an RNA genome composed of two copies of a single positive-sense RNA strand. Of the two types of HIV, type 1 and 2, type 1 (HIV-1) is the most prevalent in causing Acquired Immunodeficiency Syndrome (AIDS) worldwide.^71^ According to the latest WHO survey in 2023, approximately 39.9 million people were living with HIV, and 630,000 individuals had died owing to causes related to the virus.^72^ The symptoms of HIV vary, depending on the stage of infection. In the early stages, some people may not show any symptoms, while others may experience flu-like symptoms. As the infection progresses, the immune system gradually weakens, resulting in additional symptoms, such as swollen lymph nodes, weight loss, fever, diarrhea, and cough. Without proper treatment, HIV can lead to more severe diseases like tuberculosis, cryptococcal meningitis, severe bacterial infections, and cancers, such as lymphomas and Kaposi’s sarcoma.^73^

The virus carries a genome that encodes three lipoproteins: the Gag structural protein (group-specific antigen), the viral polymerase (Pol), and the viral envelope glycoproteins (Env). Additionally, the genome encodes other accessory proteins (Nef, Vpr, Vif, and Vpu) and regulatory proteins (Tat and Rev) involved in viral replication.^74^ HIV-1 primarily targets helper T cells and microglial cells.^75,76^ Env is the protein responsible for recognizing specific receptors of these cells and is composed of two glycoproteins: the surface glycoprotein 120 (gp120) and the transmembrane glycoprotein 41 (gp41) (Figure 1c).^77^

Env is exceptionally glycosylated, with N-glycans representing up to 50% of its total mass and covering about 70% of its surface. Each heterodimer can contain 18 to 33 potential N-glycosylation sites, the occupancy of which varies among different strains of HIV-1. This variation is influenced by mutations acquired by the virus and the host cell in which Env will be produced.^77,78^ The gp120 is the protein region with the highest amount of glycosylation, while gp41 may have 3 to 4 N-glycans.^79^ After expression of the Env protein in the host cell, it is directed to the endoplasmic reticulum (ER) for processing. At each glycosylation site, a precursor glycan (Glc3Man9GlcNAc2) is added and then modified in the Golgi apparatus, resulting in the formation of the final glycans. However, accessibility of the enzymes involved in this process is affected by steric restrictions determined by the information encoded in the viral genome, which determines the structure and position of the protein’s glycosylation sites.^77,80,81^ Most glycans that result from this processing are high-mannose N-glycans and are in the immature or unprocessed form with a Man5–9GlcNAc2 profile, either bi- or triantennary. To a lesser extent, hybrid glycans are present in the form of bi- or triantennary, while complex glycans are present in the form of bi-, tri-, or tetra-antennary, which may contain sialylation and fucosylation, the latter being predominant in the core.^79,82–87^ The profile and arrangement of glycans on the Env protein can vary considerably, but this factor and its formation are crucial for the composition of the glycan shield, which protects protein regions from immune recognition.^88,89^

A comparison of 11 Env trimers obtained from different strains, produced from different cells, and purified by distinct methods revealed that over 50% of the glycosylation sites are conserved. In the case of gp120, three conserved glycosylation sites were identified, including N156, N262, and N386, all occupied by high-mannose N-glycans. The sites present in the N332-334 and N389-N448 regions of Env vary in position among strains, but they are still occupied by high-mannose N-glycans. Additionally, the sites in the N230-N234 and N289 regions are conserved in at least 5 strains and are also occupied by high-mannose N-glycans. On the other hand, the N187, N197, N356, and N463 sites are conserved in most strains and are predominantly of the complex type. Other sites, such as N88, the N133-N139 region, N160, N241, N276, the N295-N301 region, and N339, show variation in the composition of glycans between the complex and high-mannose types. These variations may be influenced by the design of the Env construct or the type of cell that produced the protein. Among the glycosylation sites of gp41, N611, 616, 625, and N637 were identified and are conserved in all strains. However, the composition of glycans at these sites was found to be heterogeneous, varying between complex and high-mannose N-glycans.^79^

These findings provide insights into the conservation and variability of glycosylation sites in different HIV strains, as well as characteristics of the glycans present in these regions. This information is relevant for the study of immune response and the development of therapeutic strategies and vaccines.

### 2.4 Herpes Simplex (Herpesviridae)

*Herpesviridae* is a family that encompasses over 120 species of viruses, but only 9 are known to infect humans. These viruses are divided into three subfamilies, α, β, or γ, which are classified based on their genomes, structural characteristics, and biological effects.^90^ *Herpes simplex* virus (HSV) is a highly prevalent human *alphaherpesvirus* capable of causing morbidity and even death. The two subtypes are HSV-1 and HSV-2 that infect humans and can be differentiated at the genomic level and through serological tests.^91,92^ HSV-1 causes orofacial and genital infections and, in more severe cases, encephalitis. Additionally, it can lead to infectious blindness through ocular infections.^93,94^ HSV-2 predominantly causes genital infections and is more frequently associated with neonatal infections related to herpes, which can result in severe illnesses, fetal malformation, and death.^95,96^ The World Health Organization estimates that 3.7 billion people under the age of 50 have been infected with HSV-1, and 491 million people in the same age range have been infected with HSV-2. Most infected individuals do not exhibit symptoms, but depending on lifestyle during initial infection, the most common symptoms include fever, body aches, sore throat, headache, and swollen lymph nodes near the infection site. However, common symptoms of oral herpes include blisters or sores inside or around the mouth or lips, and for genital herpes, similar symptoms appear around the genital organs or anus. Symptoms are more intense during the initial episodes and gradually weaken over the course of subsequent virus outbreaks.^97,98^

HSV is an enveloped virus with an icosahedral capsid that holds a linear double-stranded DNA genome. The genome contains at least 74 genes that encode structural proteins and proteins that participate in replication processes, regulation of gene expression, assembly of new viral particles, and essential roles in the viral cycle. The capsid and envelope contain at least 20 proteins each, but 13 from the envelope are glycoproteins.^99,100^ These glycoproteins participate in the infection process, or, more specifically, binding to receptors present on the host cell membrane in the process of fusion of the viral envelope with the cell’s plasma membrane. Glycoproteins may also participate in endocytosis processes through interacting with receptors, as well as forming the glycan viral shield.^101–103^ HSV first infects epithelial cells in its lytic phase; then, infection progresses to peripheral sensory and autonomic neurons where it can establish latency and protect itself from the immune system.^104,105^

About 12 glycoproteins cover the envelope of HS: gB, gC, gD, gE, gG, gH, gI, gJ, gK, gL, gM, and gN. These have different compositions, forms, and sizes, with the vast majority presenting in a monomeric form; however, some can associate to form dimers, such as gH-gL and gE-gI. Four of these glycoproteins (gB, gD, and gH/gL) are associated with the infection process and are targets for therapeutic strategies (Figure 1d). After interacting with the host’s cell receptor, these glycoproteins interact with each other to fuse the viral envelope with the cell membrane and disperse its genome into the intracellular environment. HSV is bound to the plasma membrane by glycoprotein B (gB) through transient interactions with cell surface proteoglycans. The gB glycoprotein interacts with one of the possible receptors to mediate virus entry. The gD glycoprotein facilitates virus entry by binding to its receptors. After the binding of gD and gB, gB undergoes conformational changes through interaction with gH/gL, triggering membrane fusion.^101,106,107^

In HSV, these glycoproteins can interact with several cellular receptors. HSV has both N-glycans and mucin-like O-glycans, but it also has an unusual glycosylation profile relative to other viruses in that its envelope glycoproteins are mainly O-glycosylated, rather than N-glycosylated, and O-glycosylated elongation glycans are important for the propagation of HSV. The glycoproteins gG (8 sites), gE (16 sites), gI (4 sites), gC (12 sites), gH (4 sites), gL (4 sites), gB (21 sites), and gD (5 sites) are rich in O-glycans, while gJ, gK, gM, and gN are rich in N-glycans. Glycoproteomic analysis of HSV showed that the virus mainly expresses core 1 motifs (Neu5Acα2–3Galβ1–3GalNAcα1-O-Ser/Thr), Sialyl T antigen, T antigen, truncated Tn antigen (GalNAcα1-O-Ser/Thr), and, to a lesser extent, core 2.^108,109^

The gC glycoprotein has clusters of O-glycans and may have from one to seven units of N-acetyl-hexosamine (HexNAc), depending on the site, but it also expresses N-glycans.^109^ The gC precursor molecule has polymannosyl chains of different sizes, ranging from Man5GlcNAc to Man9GlcNAc, with a greater abundance of Man7GlcNAc and Man8GlcNAc. However, the mature gC has most of its N-glycans converted into complex glycans with a predominance of bi- and triantennary types partially sialylated on these branches.^110^ The Asn108 site expresses bi- and triantennary structures, may present fucosylation in the core and/or ends, and may contain sialylation. Asn148 presented structures of the bi-, tri-, and tetra-antennary complex type, or hybrid, to a lesser abundance, fucosylated in the nucleus and/or ends, but not sialylated. Finally, Asn-362 showed a biantennary structure with a variable degree of fucosylation and sialylation.^109^ Other glycoproteins also present N-glycosylations, such as gB, gD, gH, and gL, with complex, bi-, tri-, and tetra-antennary types with fucosylation of the core and/or end and different degrees of sialylation. They may also contain the high-mannose type with polymannosyl chains of different sizes, ranging from Man4GlcNAc to Man6GlcNAc, and finally, hybrids with polymannosyl chains, ranging from Man5GlcNAc to Man6GlcNAc, with a GlcNAc residue at the ends, but without fucosylation or sialylation.^111–115^

### 2.5 Comparative View of Viral Glycosylation

The deep analysis detailed above reveals both conserved profiles and striking divergences, which in our view, are critical to guide the future of lectin application in antiviral strategies. The dense presentation of high-mannose N-glycans is an obvious common denominator for several viruses including influenza, coronaviruses and HIV. This is not a coincidence, but a consequence of the very dense packing of glycoproteins on the viral surface which physically limits the accessibility of host glycan-processing enzymes in the endoplasmic reticulum and Golgi apparatus, resulting in incomplete trimming. This creates an “Achilles’ heel” that viruses are unable to mask.

However, significant distinctions must be noted. The heavy reliance of Herpesviruses on O-glycosylation, for instance, sets them apart and suggests that lectins with specificities beyond mannosides may be more effective. Furthermore, more subtle differences, such as the balance between high-mannose and complex glycans on SARS-CoV-2 versus MERS-CoV, or the site-specific glycan heterogeneity on influenza HA, are not mere structural footnotes. We believe these variations can dictate viral tropism, immune evasion tactics, and, importantly in the context of this review, the potential efficacy of a given lectin. Therefore, while it is unsurprising that mannose-binding lectins tend to be more potent, a one-size-fits-all approach may be insufficient. As will be seen in the next sections, great strides have been made in this area; nonetheless, the development of lectins with tailored specificities and the shift from broad-spectrum binders to precision-guided antiviral lectins are the necessary next steps.

## 3 Mechanism of Viral Entry and Antiviral Action of Lectins

Since lectins mainly act by interfering with viral infection at the entry stage, it is necessary to understand the mechanisms of viral entry into cells (Figure 2).

**Figure 2:**
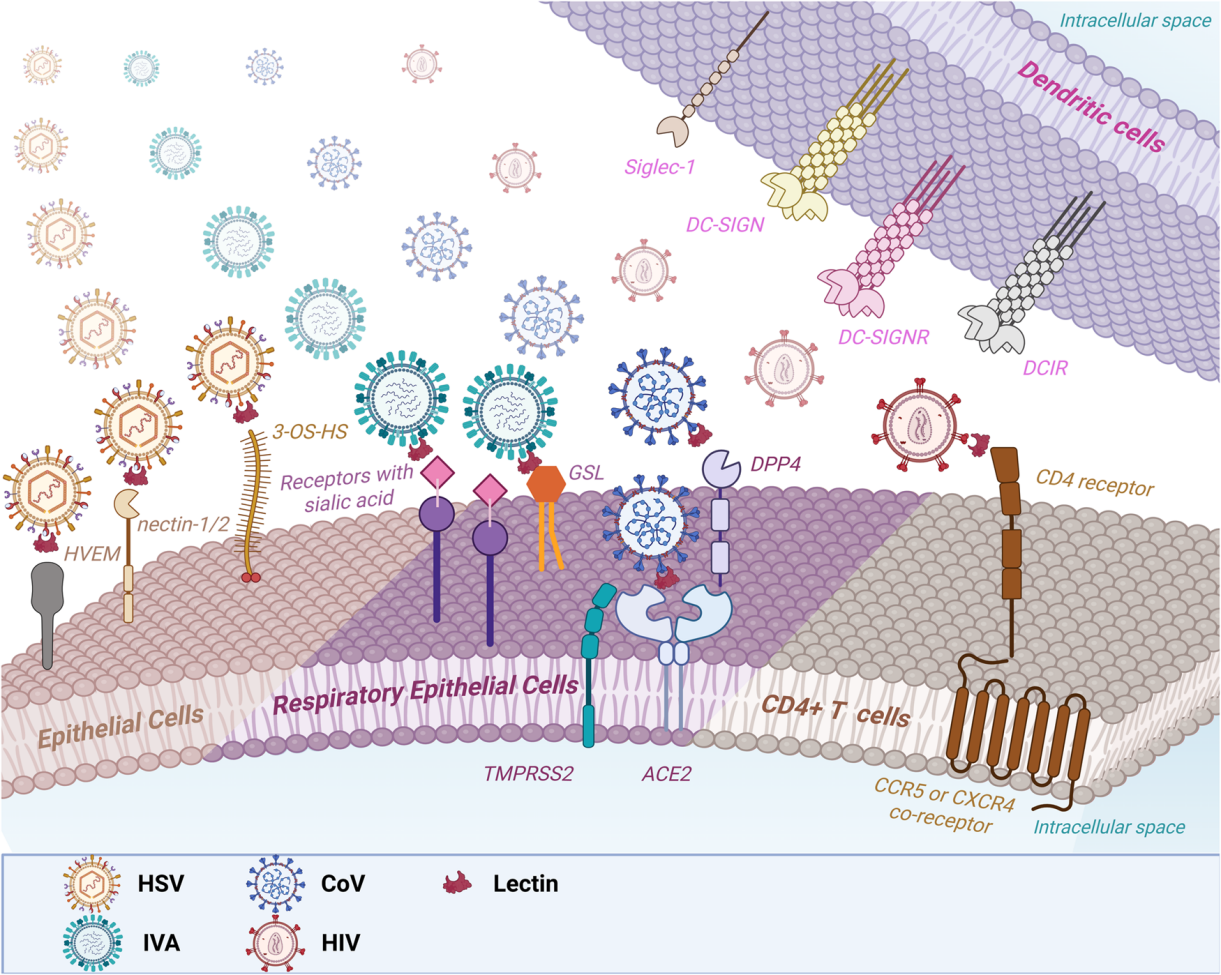
Schematic representation of viral infection by different virions (IAV, CoV, HIV, and HSV), their target cells, main receptors, and alternative pathways, in addition to lectin-mediated blocking. *Herpes simplex* virus (HSV) initially infects epithelial cells through interaction with the receptors HVEM (Herpesvirus Entry Mediator), nectin-1/-2, and 3-O-heparan sulfate (3-OS-HS). Influenza A virus (IAV) and coronaviruses (CoV), such as SARS-CoV and MERS-CoV, infect epithelial cells of the respiratory tract through interactions with receptors containing sialic acid or glycosphingolipids (GSLs), in case of the IAV, and specific receptors ACE2 (Angiotensin-Converting Enzyme 2), in the case of SARS-CoV and DPP4 (Dipeptidyl Peptidase 4), in the case of MERS-CoV. The TMPRSS2 (Transmembrane Serine Protease 2) protease facilitates the fusion of the CoV envelope with the host cell membrane, promoting viral entry. The human immunodeficiency virus (HIV) infects CD4+ T cells through interaction with the CD4 receptor and the coreceptors CCR5 or CXCR4, which are required for viral fusion and entry. In addition to these main routes, these viruses can use alternative routes of infection through interaction with receptors expressed on dendritic cells, such as DC-SIGN (Dendritic Cell-Specific Intercellular adhesion molecule-3-Grabbing Nonintegrin), DC-SIGNR (DC-SIGN-Related), DCIR (Dendritic Cell Immunoreceptor) and Siglec-1 (Sialic acid-binding Ig-like lectin 1). Lectins recognize glycans present on viral glycoproteins, blocking the interaction of viruses with their main or alternative receptors and, consequently, inhibiting cellular infection.

Influenza virus (IAV) enters host cells through the binding of hemagglutinin (HA) to sialic acid on glycoproteins and glycolipids, especially on cell surface N-glycans, with preference for α2,3 and α2,6 linkages.^116,117^ HA is produced from a precursor molecule, and after post-translational processing, it forms a trimer with a sialic acid recognition site on each protomer.^118^ Each protomer has two domains: HA1, which binds to the cellular receptor, and HA2 (stem), responsible for viral membrane fusion. The HA structure consists of a globular head, formed by the HA1 domains, and a stem, formed by part of the HA1 and HA2 domains. In viruses affecting humans, HA subtypes typically recognize α2,6-linked sialic acid terminated glycans, which are abundant on human respiratory tract cells.^119,120^ Mutations in the HA binding site can alter specificity, for example, shifting preference from α2,3-linked sialic acid (common in avian or swine hosts) to α2,6-linked forms found in humans.^121^ Recently, it was reported that IAV might also bind to nonsialylated phosphorylated high-mannose glycans via a distinct site on HA.^122^ Neuraminidase (NA) plays a crucial counterbalancing role in this process by cleaving sialic acid from mucins and cell surface receptors, preventing virion aggregation and facilitating release from infected cells and penetration of mucus.^36,39,123^ Recent studies have also indicated that O-glycans and glycosphingolipids (GSL) may contribute to viral attachment, although their contribution to internalization remains less clear.^124,125^

The spike (S) protein of SARS-CoV-1, SARS-CoV-2, and MERS-CoV mediates viral entry into target cells. While SARS-CoV-1 and SARS-CoV-2 primarily utilize angiotensin-converting enzyme 2 (ACE2) as their entry receptor, MERS-CoV employs dipeptidyl peptidase-4 (DPP4/CD26).^126–128^ Efficient entry for these CoVs requires proteolytic cleavage of the S protein by host proteases. S is synthesized as a precursor cleaved into S1 (receptor-binding) and S2 (fusion) subunits, often by furin during viral assembly or egress (especially prominent for SARS-CoV-2 due to its unique polybasic cleavage site at the S1/S2 boundary). Following receptor binding (ACE2 or DPP4 via the S1 subunit’s RBD), further cleavage within the S2 subunit (at the S2′ site) is necessary to expose the fusion peptide and trigger membrane fusion. This S2′ cleavage can be mediated by cell surface proteases like TMPRSS2 or endosomal proteases like cathepsin L (CTSL), depending on the virus and entry pathway.^129–131^ These differences in receptor usage and proteolytic activation requirements contribute to the distinct tropism and pathogenesis of these viruses.

HIV mainly infects CD4+ T lymphocytes and related myeloid cells. HIV-1 entry into these cells occurs through interaction between the viral envelope glycoprotein (Env) and specific host cell receptors.^132^ Env consists of a trimeric spike formed by three gp120/gp41 heterodimers (derived from cleavage of the gp160 precursor). The surface gp120 subunit is responsible for binding to the receptors, and the gp41 subunit is involved in membrane fusion.^133–135^ The process begins with the binding of gp120 to the primary receptor CD4 on the target cell surface. This binding induces conformational changes in gp120, exposing its binding site for a coreceptor, typically the chemokine receptors CCR5 or CXCR4 (both members of the G protein-coupled receptor family).^136^ After binding to the coreceptor, gp41 undergoes a series of conformational rearrangements that lead to the insertion of its fusion peptide (FP) into the host cell membrane. This process culminates in the refolding of gp41 into a stable six-helix bundle structure, bringing the viral and cellular membranes into close apposition and driving fusion, allowing the entry of the viral capsid into the cytoplasm.^135,137^

Herpesvirus (HSV-1 and HSV-2) entry is a complex process mediated by multiple envelope glycoproteins. Initial attachment often involves interactions between glycoproteins gB and/or gC and cell surface heparan sulfate proteoglycans. Subsequently, glycoprotein gD engages specific protein receptors, such as HVEM (a TNF receptor superfamily member), nectins (cell adhesion molecules, primarily nectin-1 for both HSV-1/2, with nectin-2 preferred by HSV-2), or specifically modified 3-O-sulfated heparan sulfate (3-OS-HS). This gD-receptor interaction triggers conformational changes propagated through the gH/gL heterodimer, ultimately activating the membrane fusion activity of gB.^101,106,138^ Furthermore, gB can directly interact with other receptors like PILRα (Paired Immunoglobulin-Like Receptor Type 2-α), influencing entry pathway choice or, in neuronal contexts, myelin-associated glycoprotein (MAG), facilitating entry into specific cell types.^139,140^

Beyond these primary receptors, secondary receptors or attachment factors, particularly cellular lectins, can be used by many of these viruses to access other routes of infection or for propagation purposes. The DC-SIGN (Dendritic Cell-Specific Intercellular adhesion molecule-3-Grabbing Nonintegrin) receptor (CD209) is a prominent example. Expressed on dendritic cells in mucosal tissues, this type II transmembrane C-type lectin has a recognition domain for high-mannose oligosaccharides. DC-SIGN binds N-glycans present on HIV gp120, Influenza HA and NA, Coronavirus S, and HSV gB, gC and gD proteins.^141–146^ This interaction promotes virus capture and transmission to susceptible target cells.^147^ The related lectin L-SIGN (DC-SIGNR/CLEC4M), expressed on certain endothelial cells (e.g., liver sinusoids, lymph nodes) and type II alveolar epithelial cells, plays a similar role.^148–150^ Other cellular lectins implicated in facilitating infection include DCIR (dendritic cell immunoreceptor), which also recognizes mannose structures on gp120, and Siglec-1 (CD169), a sialic acid-binding lectin on myeloid cells that captures HIV-1 via interaction with sialylated gangliosides incorporated into the viral membrane, facilitating trans-infection.^151–155^

Understanding these diverse entry mechanisms, particularly the roles of viral glycoproteins and their associated glycans (both N- and O-linked, as detailed in Section 2), is crucial for elucidating how exogenous lectins exert their antiviral effects, primarily by interfering with these processes. The mechanism of action of lectins involves interaction with specific glycan motifs present in the structures of viral glycoproteins, such as gp120, HA, NA, gB, gC and gD, preventing them from undergoing the structural rearrangements necessary for virus–host cell fusion (Figure 3). In addition, lectins can act by competing with cellular lectin receptors, such as DC-SIGN, DC-SIGNR, DCIR and Siglec-1, for their targets, hindering access of the virus to host cells.

**Figure 3:**
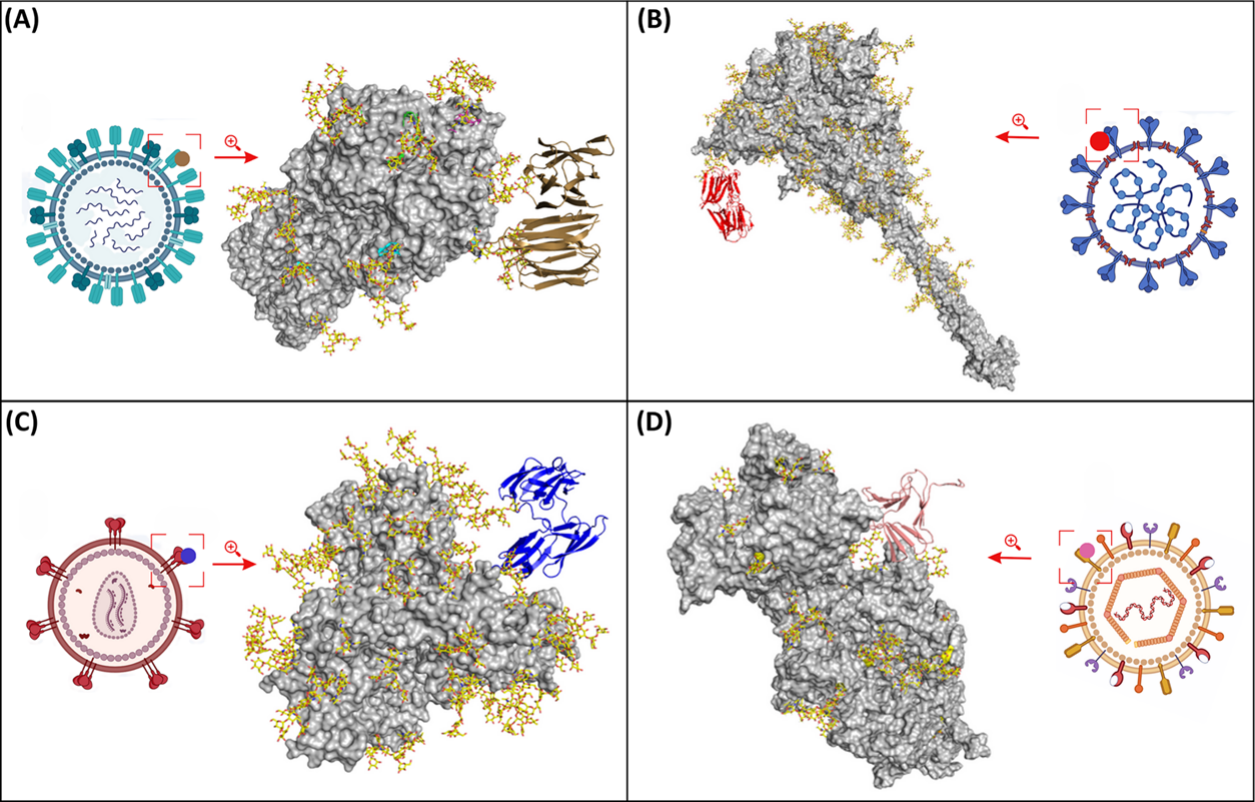
Schematic representation of the main viral surface glycoproteins targeted by lectins. (A) Influenza A virus hemagglutinin (PDB id: 4MHH) complexed with Banlec (in yellow, PDB id: 1 × 1 V); (B) SARS-CoV-2 spike glycoprotein (PDB id: 6VXX) complexed with Griffithsin (in red, PDB id: 2GUD); (C) HIV viral envelope glycoprotein (gp160) (PDB id: 5FYL) complexed with Cyanovirin-N (in blue, (PDB id: 1J4 V)); and (D) HSV-1 gB glycoprotein (PDB id: 4HSI) complexed with *Galanthus nivalis* agglutinin (in pink, PDB id: 1MSA). Glycoproteins are depicted as gray molecular surfaces, glycans as sticks with yellow carbon atoms, and lectins in cartoon representation.

## 4 Antiviral Lectins

### 4.1 Orthomyxoviridae

Numerous lectins demonstrate activity against influenza viruses. Cyanovirin-N (CV–N) (Figure 4a), from the cyanobacterium *Nostoc ellipsosporum*, inhibits both influenza A and B strains *in vitro* with EC_50_ values ranging from 0.004 to 0.04 μg/mL. Its mechanism involves binding high-mannose N-glycans on the viral HA glycoprotein.^156^

**Figure 4:**
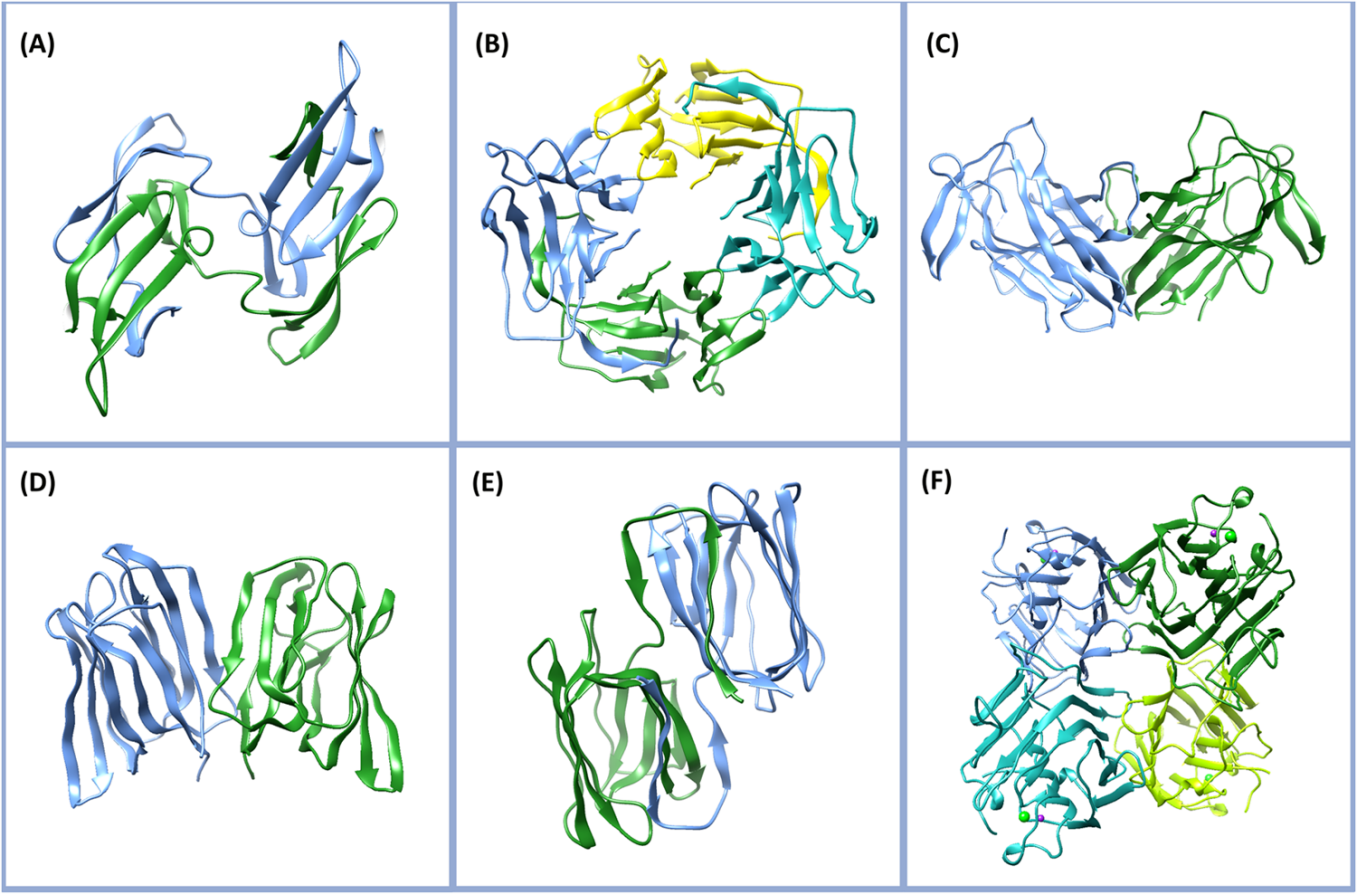
Three-dimensional structures of antiviral lectins. (A) Cyanovirin-N (CV–N; PDB ID: 1J4 V), (B) *G. nivalis* agglutinin (GNA; PDB ID: 1JPC), (C) *Nicotiana tabacum* lectin (Nictaba; PDB ID: 8QMG), (D) *Musa acuminata* lectin (BanLec; PDB ID: 5EXG), (E) *Griffithsia* sp. lectin (Griffithsin; PDB ID: 2GTY), and (F) *Canavalia brasiliensis* lectin (ConBr; PDB ID: 1AZD).

Plant lectins are a rich source of influenza inhibitors. Mannose-binding lectins from the *Amaryllidaceae* family are particularly potent. *G. nivalis* agglutinin (GNA) (Figure 4b) inhibits various influenza A strains (H1N1, H3N2) with EC_50_ values between 0.14–285 nM (H1N1) and 0.40–6.4 nM (H3N2), and influenza B strains with EC_50_ values of 0.016–0.89 nM.^157^ Similarly, *Narcissus tazetta* lectin (NTL) inhibits H1N1 replication in Madin-Darby canine kidney (MDCK) cells (EC_50_ = 4.50 μg/mL) and shows activity against H1N1 (EC_50_ = 1.33 μg/mL) and H3N2 (EC_50_ = 0.4 μg/mL), primarily targeting early viral cycle stages (likely entry).^158,159^ *Hippeastrum hybrid* agglutinin (HHA) inhibits H3N2 (EC_50_ = 2 nM) and H1N1 (EC_50_ = 540 nM) strains in MDCK cells.^160^ The N-acetylglucosamine-binding lectin from *Urtica dioica* (UDA) inhibits H1N1 (EC_50_ = 5.0–435 nM), H3N2 (EC_50_ = 5.8–83 nM), and influenza B (EC_50_ = 0.64–14 nM) strains. This activity is linked to interactions with N-glycans on the HA head, reportedly affecting virus entry and release.^157^

An engineered banana (*M. acuminata*) lectin, BanLec H84T (with reduced mitogenicity), exhibits broad *in vitro* activity against H1N1, H3N2, and H5N1 strains (EC_50_ = 0.06–11 μg/mL) and protective effects *in vivo* (rodent models). It targets high-mannose N-glycans on HA, inhibiting virus–host cell fusion.^161,162^

Inside plant lectins, legume lectins show particular promise. FRIL, from *Dolichos lablab*, inhibits diverse human and avian influenza strains (*in vitro* EC_50_ = 0.74–1.80 μg/mL) and protects mice from lethal H1N1 challenge via intranasal administration. Mechanistically, FRIL aggregates virions by binding complex-type N-glycans on HA, despite its reported preference for high-mannose structures.^163^ Other mannose/glucose-specific legume lectins, particularly *Dioclea sclerocarpa* lectin (DSL), show potent activity against influenza A and B (EC_50_ = 400–1200 pM). Related ConA-like lectins (ConBr from *C. brasiliensis*, ConM from *Canavalia maritima*, DLL from *Dioclea lasiocarpa*) are active but less potent.^164^

Algal lectins represent another significant group of influenza inhibitors. Some mannose-binding algal lectins, such as BSL (*Bryothamnium seaforthii*), HML (*Hypnea musciformis*), and MEL (*Meristiella echinocarpa*), generally exhibit weaker activity (EC_50_ > 100 μM typically), although AML (*Amansia multifida*) shows moderate potency (EC_50_ = 6–105 nM against Influenza B and H3N2).^164^ However, several other algal agglutinins display high potency, often targeting HA N-glycans. These include: *Halimeda renschii* lectin (HRL40, EC_50_ = 2.45 nM vs H3N2 in NCI-H292 cells); *Kappaphycus alvarezii* agglutinin (KAA-2, EC_50_ = 1.7–69 nM across H1N1, H3N2, Flu B in MDCK cells); *Boodlea coacta* agglutinin (BCA, EC_50_ = 19–1600 nM in MDCK cells); *Grateloupia chiangii* lectin (GCL, EC_50_ = 0.95–1.37 μM in MDCK cells); and *Eucheuma serra* agglutinin (ESA-2, EC_50_ = 0.8–35 nM in MDCK cells).^165–169^ Additionally, HBL40, a complex-type N-glycan-specific lectin from *Halimeda borneensis*, potently inhibits H3N2 (EC_50_ = 8.02 nM in NCI-H292 cells) by binding HA.^170^

From our perspective, while this wealth of *in vitro* data is compelling, the path to clinical relevance is a much steeper slope and requires a critical assessment of the *in vivo* context and the overall interest of the society as a whole for a “possibly quite expensive” anti-influenza product. It is a fact that the respiratory mucosa is densely coated with host glycoproteins that presents an important competitive barrier. We argue that lectins with the highest potential are the ones that can overcome this challenge, either through exceptionally high affinity for viral glycans or, perhaps more strategically, by targeting glycan motifs that are less common on host cells. Therefore, not only the viral glycome is important for the application, but the host glycome as well.

### 4.2 Coronaviridae

Lectins from diverse biological sources, including plants, algae, fungi, and bacteria, exhibit inhibitory activity against pathogenic coronaviruses.^171^ These proteins typically function by binding N-linked glycans, particularly high-mannose structures, on the viral spike (S) glycoprotein, thereby interfering with viral attachment or membrane fusion. Early screening studies identified mannose-binding lectins as among the most potent inhibitors of SARS-CoV, consistent with the extensive glycosylation of its S protein.^172^

A variety of plant lectins target coronavirus S proteins. UDA, specific for GlcNAc-containing glycans, interferes with SARS-CoV-1 entry by binding S protein glycans. Lectins from *Allium porrum* (leek) and *N. tabacum* (Nictaba) (Figure 4c) also inhibit CoV replication *in vitro*.^172^ Several mannose-binding plant lectins, including ConA (*Canavalia ensiformis*), GNA, and HHA, demonstrate nanomolar to low micromolar anti-SARS-CoV activity.^172–174^ (Note: ConA’s therapeutic potential is limited by mitogenicity and hepatotoxicity).^174^ Furthermore, lentil (*Lens culinaris*) agglutinin and wheat germ agglutinin (WGA) potently inhibit SARS-CoV, MERS-CoV, and SARS-CoV-2 pseudoviruses.^175^ FRIL (*D. lablab*) binds SARS-CoV-2 virions and inhibits infection *in vitro*, potentially via complex glycan interactions on spike.^163^

A particularly well-studied example is the engineered banana lectin H84T-BanLec. This point mutant retains the high-mannose binding specificity of wild-type BanLec (Figure 4d) but exhibits significantly reduced T-cell mitogenicity.^161^ H84T-BanLec potently inhibits SARS-CoV, MERS-CoV, SARS-CoV-2 (including variants), and other human CoVs at nanomolar concentrations by binding conserved N-linked glycans on the S protein.^176^ Impressively, H84T-BanLec demonstrated efficacy *in vivo*, reducing viral load in human lung organoid cultures and protecting mice against lethal MERS-CoV and SARS-CoV-2 challenge (comparable to remdesivir) when administered intranasally or systemically, without apparent toxicity.^176^

Marine algae yield some of the most potent CoV inhibitors known. Griffithsin (GRFT) (Figure 4e), from the red alga *Griffithsia* sp., is notable. GRFT possesses three identical carbohydrate-binding domains displaying high affinity for oligomannose glycans on viral glycoproteins.^177^ It exhibits potent, broad anticoronavirus activity, inhibiting SARS-CoV, MERS-CoV, and endemic human CoVs with EC_50_ values typically in the low to mid nanomolar range (e.g., ∼ 3–330 nM reported across studies).^178,179^ By engaging multiple glycan sites on the S trimer, GRFT sterically blocks receptor interaction and membrane fusion. With low cytotoxicity and demonstrated synergy with other agents (e.g., carrageenan), GRFT has advanced to preclinical and clinical development (initially for HIV, now also for SARS-CoV-2).^177,180,181^

Fungal lectins are also emerging as anti-CoV agents, often with unique specificities. *Pholiota squarrosa* lectin (PhoSL), a small lectin recognizing core α1,6-fucosylated N-glycans, binds fucosylated SARS-CoV-2 spike (including Omicron) with high affinity. It inhibits SARS-CoV-2 infection *in vitro* (submicromolar EC_50_) potentially by cross-linking spike trimers and inducing viral aggregation.^182^ Recently, hictin from shiitake mushroom (*Lentinula edodes*) extracellular vesicles was shown to potently inhibit the SARS-CoV-2 Omicron variant (EC_50_ ≈ 87 nM). Its N-terminal carbohydrate-binding domain homology suggests inhibition via spike glycan binding.^183^ While some fungal lectins may also have immunomodulatory effects, these examples highlight direct antiviral potential.

Prokaryotic mannose-binding lectins often exhibit broad antiviral spectra. CV–N targets coronaviruses. CV–N binds high-mannose oligosaccharides on the SARS-CoV-2 S protein (outside the RBD), potently blocking infection *in vitro* (inhibiting membrane fusion) and protecting animals from SARS-CoV-2 challenge.^184^ Other prokaryotic lectins like actinohivin (from Actinomadura) and microvirin (engineered cyanobacterial lectin variant), recognized primarily for anti-HIV activity via high-mannose binding, are also predicted to inhibit CoVs, though less studied in this context.^171^

The consistent powerful effect of lectins against different pathogenic coronaviruses reveal a key principle: targeting the glycan shield is an effective strategy for achieving broad-spectrum activity. In our view, this is rather essential given the continual emergence of new coronavirus variants and species. While the peptide epitopes of the spike protein mutate rapidly, the fundamental locations of glycosylation sites are more conserved. Lectins like GRFT and H84T-BanLec exploit this conservation. We believe that these lectins serve as more than just antiviral candidates; they are proof-of-concept for a platform approach against future pandemic threats. By targeting the conserved glycan shield, it may be possible to develop prophylactic or therapeutic agents that remain effective even as the virus evolves. This is a rare case where lectins have an edge over antibodies.

### 4.3 Retroviridae

A wide range of lectins, particularly those binding high-mannose glycans, exhibit potent activity against HIV by targeting the heavily glycosylated Env protein.

Mannose-binding lectins from the *Amaryllidaceae* family, previously noted for anti-influenza and anticoronavirus activity, also inhibit HIV. GNA, HHA, and Narcissus pseudonarcissus agglutinin (NPA) inhibited HIV-1 and HIV-2 infection in MT-4 cells with EC_50_ values between 0.3 and 0.7 μg/mL.^185^

Other plant lectins targeting Env glycans include: Horcolin, from barley (*Hordeum vulgare*), which inhibits HIV-1 entry (EC_50_ = 33–48 nM) via interaction with gp120 mannose residues;^186^ and Orysata, a jacalin-related mannose-specific lectin from rice (*Oryza sativa*), which inhibits HIV-1 and HIV-2 in CEM cells (EC_50_ = 1.7–5.6 μg/mL).^187^

Lectins from orchids also show significant anti-HIV activity. *Cymbidium hybrid* agglutinin (CA) and *Epipactis helleborine* agglutinin (EHA) potently inhibited HIV-1/2 in MT-4 cells (EC_50_ = 0.04–0.08 μg/mL), while *Listera ovata* agglutinin (LOA) was slightly less potent (EC_50_ = 0.1–0.3 μg/mL).^188^

The lectin UDL, noted previously for activity against other viruses, inhibits HIV with an EC_50_ of 105 nM.^188^ Similarly, *Myrianthus holstii* lectin (MHL) protects CEM-SS cells from HIV-induced cytopathicity (EC_50_ = 150 nM).^189^

Legume lectins targeting mannose structures are also active. ConA inhibits HIV-1 (EC_50_ = 2.2–2.6 μg/mL).^190,191^ ConA-like lectins, mentioned previously for anti-influenza activity (Section 4.1), show varying potencies against HIV-1/2: DSL (*D. sclerocarpa*) is most potent (EC_50_ = 20 nM vs HIV-1; 88 nM vs HIV-2), followed by DLL (*D. lasiocarpa*) (EC_50_ = 31 nM/89 nM), ConM (*C. maritima*) (EC_50_ = 65 nM/108 nM), and ConBr (EC_50_ = 73 nM/137 nM, Figure 4f).^164^

Algal lectins include some of the most potent HIV inhibitors. GRFT shows exceptional activity against diverse HIV-1 variants (EC_50_ = 0.01–0.07 nM in TZM-bl/CEM cells). Its high potency relies on multivalent binding to oligomannose glycans on gp120 via its dimeric structure.^192^ *K. alvarezii* agglutinin (KAA-2) inhibits HIV-1 entry (EC_50_ = 7.3–12.9 nM) via gp120 interaction.^193^ Similarly, *Boodlea coacta* lectin (BCL, also known as BCA) inhibits HIV-1 in MT-4 cells (EC_50_ = 8.2 nM).^167^

Cyanobacteria are another source of highly potent mannose-binding anti-HIV lectins. Microvirin and its engineered multivalent variant (LUMS1) inhibit HIV-1 entry (EC_50_ = 2–12 nM and ∼37 nM, respectively).^194–196^ A related lectin from *Microcystis viridis* inhibits Env-mediated cell fusion (EC_50_ = 30 nM).^197^ CV–N with broad activity noted earlier, remains one of the most potent anti-HIV lectins (EC_50_ ≈ 0.1 nM). It inhibits virus–cell fusion by binding high-mannose glycans on both gp120 and gp41.^198–201^ *Scytonema varium* lectin (SVN) also exhibits high potency (EC_50_= 0.3 nM) against various HIV-1 strains by binding Env glycans.^202^ *Oscillatoria agardhii* agglutinin (OAA) inhibits HIV-1 in MT4 cells (EC_50_ = 45 nM).^203^

While the threats posed by respiratory viruses are acute, we argue that the application of lectins against HIV addresses a unique and profound societal demand. HIV/AIDS remains a chronic, incurable global crisis, and the four-decade-long search for a vaccine has been unsuccessful. This creates a specific, unmet need for novel prevention modalities, particularly women-controlled topical microbicides to block sexual transmission. In our view, lectins are almost perfectly suited for this role. Their ability to potently and broadly neutralize the virus makes them ideal candidates for gels, films, or rings that can be applied at the site of transmission. Indeed, it is the anti-HIV application that has pioneered the clinical translation of lectins, with agents like GRFT advancing through Phase 1 trials as a microbicide. This sustained effort against a chronic pandemic provides a distinct but equally critical public health focus compared to the need for rapid-response agents against acute viral outbreaks. The lessons learned from developing anti-HIV lectins, from protein engineering to formulation and clinical trial design, provide a roadmap for the entire field.

### 4.4 Herpesviridae

Several lectins, particularly mannose-binding ones, exhibit potent activity against Herpesviridae members. GRFT inhibits HSV-2 infection *in vitro* (EC_50_ ≈ 230 nM) by binding viral glycoproteins like gD and blocking entry. Applied vaginally with carrageenan, GRFT synergistically reduced HSV-2 infection in a mouse model.^204^ CV–N potently inhibits HSV-1 entry (EC_50_ ≈ 2.3 μg/mL in Vero cells when added during infection) and cell–cell fusion mediated by HSV glycoproteins, acting independently of the specific gD receptor pathway. Its efficacy is reduced if added postentry (EC_50_ ≈ 30 μg/mL). *In vivo*, intraperitoneal administration of CV–N (5–10 mg/kg) delayed HSV-1 encephalitis onset and prolonged survival in mice.^205,206^

Plant-derived lectins also target HSV. BanLec displays virucidal activity and inhibits HSV-1 (EC_50_ ≈ 16.0 μg/mL, SI = 10.8 in Vero cells) and HSV-2 (EC_50_ ≈ 67.7 μg/mL, SI = 2.6) infectivity, possibly via virion aggregation.^207^ Differential activity is seen with GlcNAc-binding lectins: Nictaba potently inhibits HSV-1 (EC_50_ ≈ 263 nM) and HSV-2 (EC_50_ ≈ 53 nM) in human embryonic lung (HEL) fibroblasts, whereas UDA is inactive against HSV-1 (>11 μM) and much less potent against HSV-2 (EC_50_ ≈ 1.3 μM), likely reflecting differences in binding accessibility or affinity for specific HSV glycan structures.^160^ Classic mannose-binding Amaryllidaceae lectins, like GNA and HHA, also inhibit herpesviruses at submicromolar levels, while lectins specific for other motifs (e.g., galactose) are generally inactive.^188^

Activity extends beyond HSV. Varicella-zoster virus (VZV) is inhibited by Nictaba *in vitro* (EC_50_ ≈ 0.13 μM in HEL cells).^160^ Human herpesvirus 6 (HHV-6) infection of peripheral blood mononuclear cells and glycoprotein-mediated cell–cell fusion are potently blocked by CV–N (∼100 nM for complete suppression).^208^ Human cytomegalovirus (HCMV) replication is inhibited by GNA, HHA, and related mannose-specific lectins (EC_50_ ≈ 1.6 μg/mL in cell culture), presumably by binding N-glycans on essential envelope glycoproteins like gB and gH.^185^ Supporting this, recombinant human mannose-binding lectin (MBL), an innate immune protein, neutralizes HCMV infection in a mannose-dependent manner, reducing viral entry and spread.^209^

Data on exogenous lectin activity against Epstein–Barr virus (EBV) are limited. However, its major envelope glycoprotein gp350 is exceptionally heavily glycosylated, primarily with complex-type N- and O-linked chains.^210^ While this complex profile might limit binding by strictly oligomannose-specific lectins, incompletely processed glycans or GlcNAc-terminating structures could offer potential binding sites. Furthermore, genetic associations between human MBL polymorphisms and susceptibility to EBV (and HSV) infections suggest endogenous lectin interactions play a role *in vivo*.^211^ A summary of the activities can be seen in Table 1.

**Table 1:** Lectins with Antiviral Activity against Enveloped Viruses from Different Families^a^

source/lectin	virus	EC50/IC50	experimental model	mechanism of action
*Canavalia ensiformis*/ConA	HIV-1, SARS-CoV	2.2–2.6 μg/mL (HIV-1/SARS-CoV)	cell-based (MT-4) pseudovirus assay	binds mannose-rich glycans on viral envelopes; induces virion aggregation and inhibits entry^190^
*Dolichos lablab*/FRIL	Influenza, SARS-CoV-2	0.74–1.80 μg/mL (SARS-CoV-2)	cell-based (MDCK) *In vivo* (animal model)	multivalent binding to complex N-glycans on viral glycoproteins; blocks host receptor interaction^163^
*Eucheuma serra*/ESA-2	Influenza A (H1N1)	0.8–34.6 nM (Influenza A)	cell-based (MDCK)	binds high-mannose glycans on influenza HA; inhibits receptor interaction and entry.^169^
*Galanthus nivalis*/GNA	Influenza A, HIV, HCMV, HSV	0.14–285 nM ((Influenza); 0.3–0.7 μg/mL - HIV/HSV/HCMV)	cell-based (MT-4) cell-based (HEL)	mannose-binding; Inhibition of viral entry^185^
*Griffithsia sp*./GRFT	SARS-CoV, MERS-CoV, HIV-1, HSV-2	0.0032–0.33 μM (SARS/MERS); 0.01–0.07 nM (HIV-1); 230 nM (HSV-2)	cell-based (CEM) cell-based (MDCK) *in vivo* (animal model)	multivalent glycan binding; inhibits fusion^178,192^
Human (serum)/MBL	HCMV	10 μg/mL (HCMV)	cell-based (HEL)	N-glycan binding; inhibits spread^209^
*Kappaphycus alvarezii*/KAA-2	HIV-1, H3N2, Influenza B	7.3–12.9 nM (HIV-1); 1.7–68.6 nM (Influenza)	cell-based (TZM-bl) cell-based (MDCK)	binds high-mannose N-glycans on viral envelope proteins (e.g., HA, gp120); blocks viral entry^166,193^
*Lentinula edodes*/Shictin	SARS-CoV-2 (Omicron)	∼87 nM (SARS-CoV-2)	cell-free biochemical assay	glycan binding; entry inhibition^183^
*Microcystis aeruginosa*/MVN	HIV-1	2–12 nM (HIV-1)	cell-based (TZM-bl)	gp120 binding; blocks entry^194^
*Musa acuminata*/BanLec H84T	Influenza A (H1N1–H5N1), SARS-CoV-2, HSV	0.06–11 μg/mL (Influenza A); 16–67.7 μg/mL (SARS-CoV-2)	cell-based (MDCK) *In vivo* (animal model)	binds N-glycans; inhibits fusion^161,207^
*Narcissus tazetta*/NTL	Influenza A (H1N1–H5N1)	1.33–>20 μg/mL (Influenza A)	cell-based (MDCK)	early replication inhibitor^158,159^
*Nicotiana tabacum*/Nictaba	HSV-1, HSV-2, VZV	263 nM (HSV-1); 53 nM (HSV-2); 130 nM (VZV)	cell-based (HEL)	binding to oligomannosidic glycans on viral glycoproteins; inhibits entry.^160^
*Nostoc ellipsosporum*/CV–N	Influenza A/B, SARS-CoV-2, HIV, HSV	0.004–0.04 μg/mL (HIV); 0.1 nM (SARS-CoV-2); 2.3 μg/mL (HSV)	cell-based (CEM) cell-based (MDCK) cell-based (Vero) *in vivo* (animal model)	high-mannose N-glycan binding; blocks entry^156,206^
*Pholiota squarrosa*/PhoSL	SARS-CoV-2 (Omicron)	submicromolar (SARS-CoV-2)	in vitro	binds α1,6-fucose; aggregation of spike protein^182^
*Urtica dioica*/UDA	Influenza A, SARS-CoV, HIV, HSV-2	105 nM (HIV); 1.3 μM (HSV-2)	cell-based (MT-4) cell-based (Vero)	GlcNAc-binding; blocks penetration^172^

a EC_50_/IC_50_ = effective or inhibitory concentration to reduce viral infection by 50%. MDCK = Madin-Darby Canine Kidney cells; Vero = African green monkey kidney cells; CEM/MT-4/HEL = human-derived cell lines used in antiviral assays; TZM-bl = HeLa cells with CD4/CCR5/CXCR4 expression.

In our opinion, the unique glycobiology of herpesviruses, particularly their extensive use of *O*-linked glycans, marks this family as an underexplored frontier for lectin-based antivirals. While many successful antiviral lectins are mannose-specific, this specificity may be suboptimal for herpesviruses, whose surfaces are rich in mucin-like domains decorated with O-glycans (e.g., core 1 and 2 structures). The strong activity of Nictaba might be due to its ability to recognize terminal GlcNAc on some of these structures. We propose that a stronger effort should be made to screen for or engineer lectins that specifically target common O-glycan motifs like the Tn (GalNAc) or T (Gal-β1,3-GalNAc) antigens. Targeting these O-glycans could provide a novel and highly specific mechanism for inhibiting herpesvirus infection, moving beyond the well-trodden path of mannose-binding lectins.

Unlike traditional antiviral approaches, such as neuraminidase inhibitors, protease inhibitors, or neutralizing monoclonal antibodies, lectins act by recognizing glycan motifs that are often conserved across virus families. This distinction provides a unique advantage: while protein-based epitopes are prone to escape mutations, particularly in RNA viruses, the core structures of *N*-linked or *O*-linked glycans remain relatively stable. Furthermore, lectins operate independently of the precise amino acid sequence, allowing them to maintain efficacy even against emerging variants. Compared to antibodies, lectins are smaller, often more thermostable, and can be produced in a wider range of systems, including microbial and plant-based expression platforms. These properties not only reduce production costs but also make them suitable for topical and mucosal applications where antibody stability may be compromised. Importantly, lectins such as GRFT and H84T-BanLec have demonstrated potent in vivo efficacy across multiple virus families, suggesting that glycan-targeting antivirals may serve as a broad-spectrum and mutation-resilient alternative to existing modalities.

## 5 Biotechnological Applications of Lectins

Beyond their direct antiviral activity, advances in molecular engineering and materials science are expanding the biotechnological applications of lectins (Figure 5).

**Figure 5:**
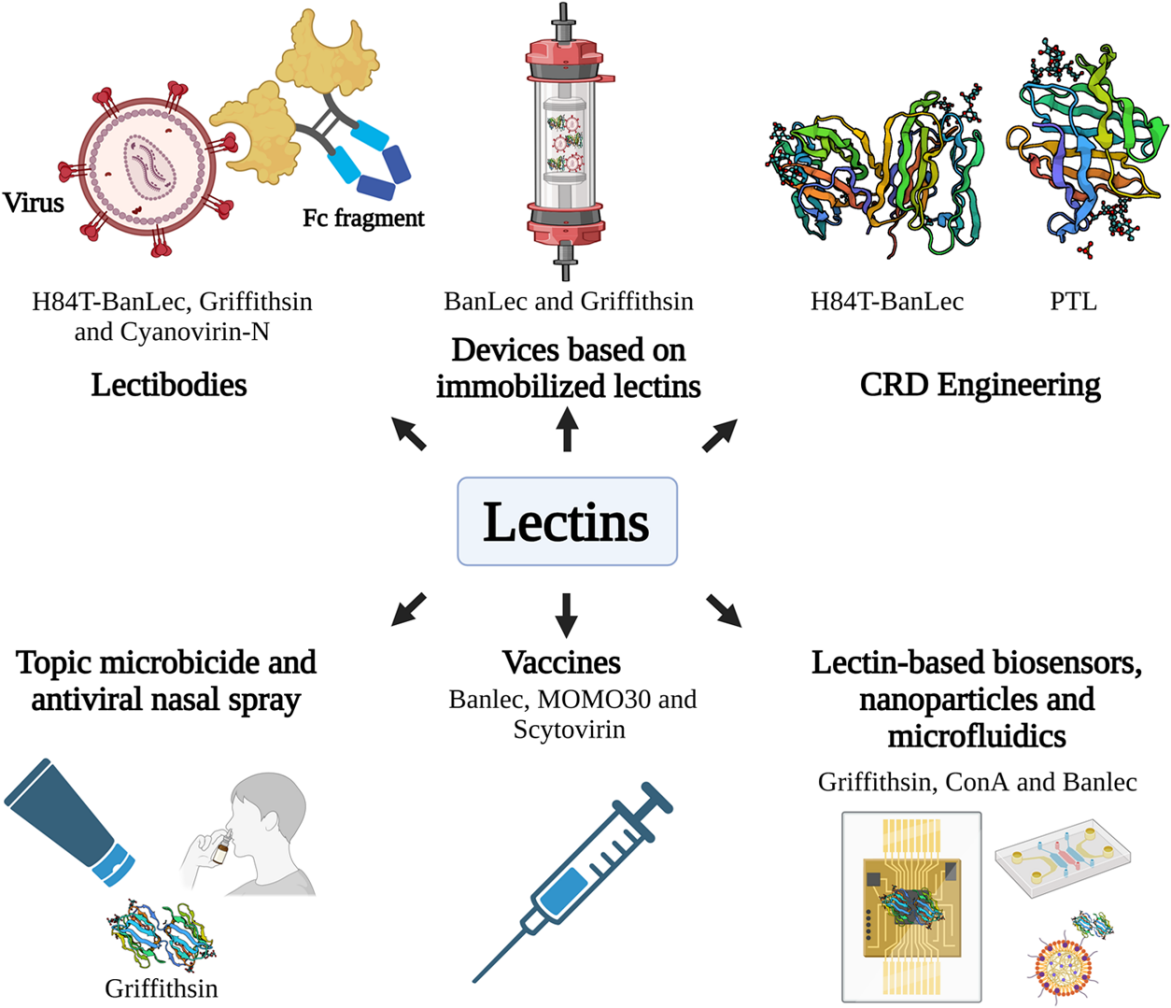
Biotechnological applications of lectins in therapeutic and diagnostic approaches against viruses.

The capacity of lectins to recognize specific glycosylation motifs makes them excellent candidates for the development of diagnostic tools. Unlike conventional antibodies, lectins can be immobilized on sensor platforms to detect viral glycoproteins with high sensitivity and stability. For example, ConA and WGA have been employed in electrochemical biosensors for detecting arboviruses and influenza envelope proteins, while NPL and GRFT have demonstrated utility in ELISA and point-of-care formats for HIV and coronaviruses. These biosensors offer several advantages, including rapid response times, label-free detection, and resilience to environmental fluctuations. Additionally, microarray-based technologies and lectin conjugation strategies further enhance signal transduction and enable multiplexed viral detection. Current developments involving optical, electrochemical, and microfluidic detection systems illustrate the versatility of lectin-based platforms and support their application in emerging diagnostic technologies for viral infections.

One major strategy enhances therapeutic potential through fusion proteins, notably “lectibodies,” which combine a lectin’s carbohydrate-binding domain with an antibody Fc region. As introduced earlier (Section 1), this design aims to improve pharmacokinetics and stability while enabling Fc-mediated effector functions (e.g., ADCC, CDC).^7,212^ Direct protein engineering also refines lectin properties. Key goals include enhancing antiviral potency or breadth, improving stability, and mitigating adverse effects like toxicity or mitogenicity.^213^ A prominent example is H84T-BanLec, engineered to reduce mitogenicity while retaining broad antiviral efficacy.^161,162^ Site-specific engineering of other lectins, like *Pseudomonas taiwanensis* lectin (PTL), has also improved antiviral activity.^214^

The specific glycan recognition of lectins makes them valuable diagnostic and research tools. They enable detection and quantification of viral glycoproteins in various assay formats. For instance, ConA is used to detect arbovirus glycoproteins, and NPL has been applied in ELISAs to quantify HIV gp120.^215,216^ Furthermore, lectins immobilized on diverse platforms (e.g., electrochemical, plasmonic, microfluidic) form the basis of sensitive biosensors for rapid viral detection.^217^ As research tools, lectins remain critical for characterizing viral glycosylation and probing virus–host interactions.^218^

Lectin properties are also exploited in materials science and other applications. Lectin immobilization onto surfaces is being explored for passive antiviral strategies. Patents describe using mannose-binding lectins (including BanLec) on filters, personal protective equipment, or extracorporeal devices to capture and neutralize virions, potentially reducing transmission.^219,220^ Immunomodulatory properties also allow potential use as vaccine adjuvants, as some lectins can enhance immune responses to coadministered antigens (The approved recombinant influenza HA vaccine utilizes the virus’s own receptor-binding capacity, not typically an exogenous adjuvant lectin).^221,222^ Separately, traditional medicine utilizes certain lectin-containing plants (e.g., bitter melon, *Momordica balsamina*, containing MOMO30) for purported antiviral effects, with some preliminary clinical observations reported for HIV, though rigorous validation is needed.^223,224^

Clinical translation of lectin antivirals is advancing slowly but steadily. GRFT completed Phase 1 trials as a topical microbicide for HIV (showing good safety), and an engineered variant (Q-Grft) is in trials as a nasal spray against SARS-CoV-2.^177,181,225,226^ Some positive outcomes were reported for MOMO30-containing tea in HIV patients.^223^ Numerous preclinical studies, involving lectins like H84T-BanLec, FRIL, and UDA against various viruses (as detailed in Section 4), provide further validation of therapeutic potential.^162,163,227^ However, bridging the gap to approved therapeutics remains challenging, and real-world efficacy data, particularly for commercial products or traditional remedies, is scarce.

Despite promising applications, several key challenges hinder widespread lectin-based antiviral development, echoing concerns raised earlier. These can be broadly categorized as

1. Biological/Safety Issues: Potential immunogenicity, off-target toxicity (including mitogenicity), and hemagglutination activity can limit systemic administration and necessitate careful engineering or targeted delivery strategies.^161,228,229^

2. Viral Factors: The high mutation rate of RNA viruses can lead to altered glycosylation patterns, potentially reducing lectin efficacy and facilitating viral escape. Strategies focus on targeting conserved glycan sites or using combinations.^230,231^

3. Production and Delivery: Scalable, cost-effective production remains a hurdle, though recombinant expression systems offer solutions. Optimizing administration routes (e.g., topical/mucosal vs systemic) is critical for maximizing efficacy and minimizing toxicity.^232–235^

In the context of the above considerations, a clear overview of the diverse uses and ongoing challenges in this field is given in Table 2 below. It summarizes the main biotechnological applications of lectins, along with relevant examples and future perspectives

**Table 2:** Biotechnological Applications of Lectins in Antiviral Research

**application**	**description**	**examples**	**challenges/future perspectives**	**techniques**
• **therapies**	lectins inhibit viral entry and are evaluated in clinical settings for antiviral effects.	Griffithsin (HIV, SARS-CoV-2), H84T BanLec (influenza), FRIL (H1N1), Q-Grft (nasal spray), MOMO30 (*Momordica balsamina* tea in HIV)	toxicity, immunogenicity, hemagglutination, limited trials, formulation optimization	protein engineering, recombinant expression, biosensors, clinical trials, glycosylation analysis
• **clinical trials**				
• **protein engineering**	rational modification of lectins to enhance antiviral activity and reduce side effects.	H84T-BanLec (reduced mitogenicity), modified PTL (enhanced antiviral activity), engineered lectins (e.g., mGRFT)	balancing efficacy and safety, maintaining stability after mutations	site-directed mutagenesis, protein engineering, glycan array, biosensors
**• lectin modification**				
**• diagnostics**	lectins used for detecting viral glycoproteins and preventing viral infections in various environments.	ConA (arboviruses), NPL (HIV-gp120), BanLec in PPE, GRFT (air filtration), biosensors with immobilized lectins	sensitivity, specificity, standardization, stability in environments	ELISA, biosensor integration, glycan array, microfluidics, optical biosensors (SPR)
**• prevention**				
**• adjuvants**	lectins can enhance immune responses and are used in traditional phytotherapy for antiviral effects.	recombinant hemagglutinin in vaccines, *Musa acuminata* (BanLec) in HIV, *Momordica balsamina* (MOMO30), Scytovirin (HSV-2)	safety, unwanted immune response, scientific validation	vaccine formulation, ethnobotanical surveys, glycosylation profiling, immune recruitment assays
**• phytotherapy**				
**• nanotechnology**	nanoparticle conjugation and microfluidics for enhanced viral capture and point-of-care diagnostics.	ConA, GRFT, WGA (nanoparticles), GRFT-functionalized microfluidic devices	signal enhancement, capture efficiency, integration in diagnostics	nanoparticle conjugation, microfluidic device functionalization, biosensor technology
**• functionalized systems**				
• **chimeric lectins**	hybrid lectins and lectin-antibody fusion proteins to enhance antiviral potency and immune responses.	H84T-BanLec, CV–N, GRFT (multidomain), Avaren-Fc (AvFc), GRFT-Fc, Lectibodies (Fc-fused)	potency vs specificity, immune activation without adverse responses	Fc fusion, protein engineering, hybrid lectins, immune recruitment assays, recombinant expression
**• lectibodies**				
**• prevention**	immobilized lectins capture and inactivate viruses in environmental settings or devices.	BanLec (PPE), air filtration systems, extracorporeal devices (GRFT)	stability of immobilized lectins, material compatibility, large-scale application	surface immobilization, air filtration systems, extracorporeal devices
**• filtration**				

## 6 Conclusions and Future Directions

This review confirms the established versatility of lectins as potent, glycan-targeting agents with dual utility in antiviral therapy and diagnostics. The principle is clear: the glycosylation of enveloped viruses, while providing camouflage from the immune system, creates conserved vulnerabilities that can be exploited. However, a realistic analysis of the field demands that we move beyond celebrating in vitro potencies and confront the huge barriers that have largely confined these remarkable proteins to the laboratory. The path from a promising molecule to a clinically approved drug is full of challenges, and for lectins, these are significant.

The reality is that significant hurdles remain. First, for most unmodified lectins, the inherent risks of immunogenicity and off-target toxicity make systemic administration a high-risk proposition. The very mechanism that makes them effective, binding to common glycans, also makes them prone to interacting with host cells, potentially leading to mitogenicity or cytotoxicity. Second, the economics of production are daunting. Scaling up the GMP-compliant manufacturing of a recombinant biologic is a costly and complex endeavor that represents a major commercial bottleneck. Finally, the specter of viral escape looms large; the high mutation rate of RNA viruses means that any monotherapy targeting a limited number of glycan sites is vulnerable to becoming obsolete. These are not minor issues; they are the primary reasons why, despite decades of research, no lectin-based antiviral has completed late-stage clinical trials.

Yet, a pragmatic, two-tiered path forward exists. The most direct route to the clinic, in our view, involves focusing on applications where the therapeutic index is most favorable. Topical and mucosal delivery for prophylaxis or treatment at the portal of entry remains the lowest-hanging fruit. The development of lectin-based vaginal microbicides for HIV or intranasal sprays for respiratory viruses bypasses the most severe systemic safety concerns and represents the greatest chance for near-term success. Simultaneously, the quest for safe and effective systemic agents should not be abandoned but pursued through the lens of advanced bioengineering. The creation of ″lectibodies″, fusing lectin domains to antibody Fc regions, is a key strategy aimed directly at solving the systemic challenge by improving pharmacokinetic profiles and leveraging Fc-mediated effector functions. This, along with innovations in nanotechnology for targeted delivery and the development of extracorporeal devices for blood purification, represents the ambitious frontier of the field. These advanced applications require overcoming a higher safety and engineering bar, but they hold the promise of treating established, disseminated infections.

To enable either path, the field must fully embrace a paradigm of rational engineering over serendipitous discovery. The goal is no longer just finding the next potent lectin but designing biologics with superior safety profiles. To counter viral resistance, the strategy must be combinatorial, using either lectin ″cocktails″ or, more elegantly, multispecific fusion proteins engineered to engage multiple glycan epitopes simultaneously. The continued development of lectin-based diagnostics runs parallel to these efforts, representing a more accessible route to market that provides invaluable tools for surveillance and disease management.

In conclusion, the promise of lectins is real, but it must be pursued with strategic discipline. A tiered approach, prioritizing topical applications for immediate clinical translation while investing in the sophisticated bioengineering required for safe systemic use, offers the most realistic path forward. The bridge from lab to clinic for lectins will not be built on naive optimizm, but on a pragmatic, multifront design strategy aimed at solving specific, critical public health needs.

## Data Availability

The data are available from the corresponding authors upon reasonable request. No registration or data use agreement is required for access.
